# First Results From a Calibrated Network of Low‐Cost PM_2.5_ Monitors in Mombasa, Kenya Show Exceedance of Healthy Guidelines

**DOI:** 10.1029/2024GH001049

**Published:** 2024-09-21

**Authors:** M. N. Njeru, E. Mwangi, M. J. Gatari, M. I. Kaniu, J. Kanyeria, G. Raheja, D. M. Westervelt

**Affiliations:** ^1^ Institute of Nuclear Science and Technology University of Nairobi Nairobi Kenya; ^2^ Department of Physics University of Nairobi Nairobi Kenya; ^3^ Institute of Energy and Environmental Technology Jomo Kenyatta University of Agriculture and Technology Nairobi Kenya; ^4^ Department of Earth and Environmental Sciences Columbia University New York NY USA; ^5^ Lamont‐Doherty Earth Observatory of Columbia University New York NY USA

**Keywords:** air sensors, particulate matter, Kenya

## Abstract

The paucity of fine particulate matter (PM_2.5_) measurements limits estimates of air pollution mortality in Sub‐Saharan Africa. Well calibrated low‐cost sensors can provide reliable data especially where reference monitors are unavailable. We evaluate the performance of Clarity Node‐S PM monitors against a Tapered element oscillating microbalance (TEOM) 1400a and develop a calibration model in Mombasa, Kenya's second largest city. As‐reported Clarity Node‐S data from January 2023 through April 2023 was moderately correlated with the TEOM‐1400a measurements (*R* ^2^ = 0.61) and exhibited a mean absolute error (MAE) of 7.03 μg m^−3^. Employing three calibration models, namely, multiple linear regression (MLR), Gaussian mixture regression and random forest (RF) decreased the MAE to 4.28, 3.93, and 4.40 μg m^−3^ respectively. The *R* ^2^ value improved to 0.63 for the MLR model but all other models registered a decrease (*R* ^2^ = 0.44 and 0.60 respectively). Applying the correction factor to a five‐sensor network in Mombasa that was operated between July 2021 and July 2022 gave insights to the air quality in the city. The average daily concentrations of PM_2.5_ within the city ranged from 12 to 18 μg m^−3^. The concentrations exceeded the WHO daily PM_2.5_ limits more than 50% of the time, in particular at the sites nearby frequent industrial activity. Higher averages were observed during the dry and cold seasons and during early morning and evening periods of high activity. These results represent some of the first air quality monitoring measurements in Mombasa and highlight the need for more study.

## Key Points

- Mean daily PM_2.5_ concentrations in Mombasa ranged from 12 to 18 μg m^−3^ depending on site location and time of year
- Daily PM_2.5_ concentrations were higher during the dry seasons, early morning and afternoon, and lower during the wet seasons
- Sites nearby frequent industrial activity exceeded the WHO daily limits of PM_2.5_ more than 50% of the time

## 1 Introduction

Air pollution poses a considerable threat on world health, with its most pronounced impact felt in low‐ and middle‐ income countries (LMICs). Currently ranking fourth among the leading causes of global morbidity and mortality, it closely trails high blood pressure, smoking and unhealthy diets (Hoffmann et al., 2021). The gravity of the situation is underscored by epidemiological studies associating about 6.5 million premature deaths and 6 million preterm births globally each year to air pollution (Ghosh et al., 2021; McDuffie et al., 2021). These statistics highlight the imperative to prioritize interventions that tackle the diverse health risks posed by air pollution.

Fine particulate matter (PM), known as PM_2.5_, stands out as the most hazardous among major air pollutants. These particles are easily respirable and exhibit a propensity to deposit in the pulmonary region based on their size (Dharaiya et al., 2023). Controlling PM pollution is a key focus of national and local government bodies in many countries (e.g., the Environmental Protection Agency in the United States) and is historically measured using certified reference methods, with a high degree of accuracy and precision. Devices fitting this description are normally filter‐based methods like high volume samplers, though near real time monitoring methods like beta attenuation monitors and tapered element oscillating microbalance (TEOM) are also certified and used in air quality management (Ghamari et al., 2022; Hagan & Kroll, 2020). While these meet most legal requirements, equipping and maintaining air quality stations with such monitors can be a financial burden and often results in relatively sparse monitoring. In a complex urban environment, for instance, a single reference monitor costing more than $10,000 cannot give information about localized variations that are important for protecting health. Depending on deployment characteristics, a single reference monitor may only represent tens or hundreds km^2^ by area and inform pollution in highly specific geographies (Alfano et al., 2020; Levy Zamora et al., 2019).

Fortunately, there has been a new paradigm shift in conventional PM monitoring with the advent of low‐cost sensor systems. These devices, primarily portable optical particle counters or nephelometers, operate based on the principle of light scattering to infer the PM number concentration, which can then be converted to mass concentration assuming a particle density and shape. Priced between $150 to $3,000, these devices offer a cost‐effective solution to capture spatiotemporal variability, enabling high‐density near real‐time air quality monitoring (Feenstra et al., 2019; Zimmerman et al., 2018). Recent work has shown that low‐cost air quality sensors, especially when carefully calibrated, can be extremely powerful in revealing air quality levels and sources of air pollution (Amegah, 2018; Giordano et al., 2021; McFarlane, Isevulambire, et al., 2021; McFarlane, Raheja, et al., 2021; Okure et al., 2022; Raheja et al., 2022, 2023; Subramanian & Garland, 2021; Westervelt et al., 2024). An outstanding issue remains data quality, though the strengths and weaknesses of these devices have been well‐characterized recently (Hagan & Kroll, 2020; Jayaratne et al., 2018; Molina Rueda et al., 2023; Ouimette et al., 2022; Tryner et al., 2020).

For LMICs like Kenya, where adequate monitoring and scientific information are lacking, the potential of low‐cost sensors cannot be overstated. With only a few reference monitors and a few sensors reporting air quality data, primarily concentrated in the capital, Nairobi, there is a pressing need for comprehensive monitoring in other regions of the country. Previous studies on air quality in Mombasa are few (Simiyu et al., 2018; Yussuf et al., 2023) and have only relied on simulated model output, for example, from the Modern‐Era Retrospective analysis for Research and Applications version 2 reanalysis (MERRA‐2). This work therefore presents, to our knowledge, the first surface observations of PM_2.5_ in the city of Mombasa, the second‐largest city in Kenya with a population of about 3.5 million and a major port city, and represents some of the first dedicated air quality research in this area.

## 2 Materials and Methods

### 2.1 Sampling Locations

Mombasa is the second largest city in Kenya and lies on the southeast of the Kenyan coast within coordinates (3°80′, 4°10′S and 39°60′, 39°80′E). The city has an area of 295 km^2^ with an increasing number of inhabitants at more than 3.5 million (KNBS, 2019). It is arguably the largest port in East Africa and plays a pivotal role in trade in the region. It is home to several manufacturing and processing industries including iron smelting, steel rolling mills, cement mining and oil companies. Mombasa is also an iconic tourist destination with clusters of sandy beaches and World Heritage sites (KPA, 2017).

Despite its economic significance, Mombasa faces understudied environmental challenges, particularly in terms of air quality. Potential anthropogenic sources of pollution include operation of minibuses (Matatus), motorized tricycles (Tuk Tuks), cargo ships, haulage trucks, container handling equipment, thermal power plants, cement factories, and the burning of open and biomass fuels. The combination of industrial activities, transportation, and tourism makes Mombasa a complex urban environment susceptible to air quality issues.

**Figure 1:**
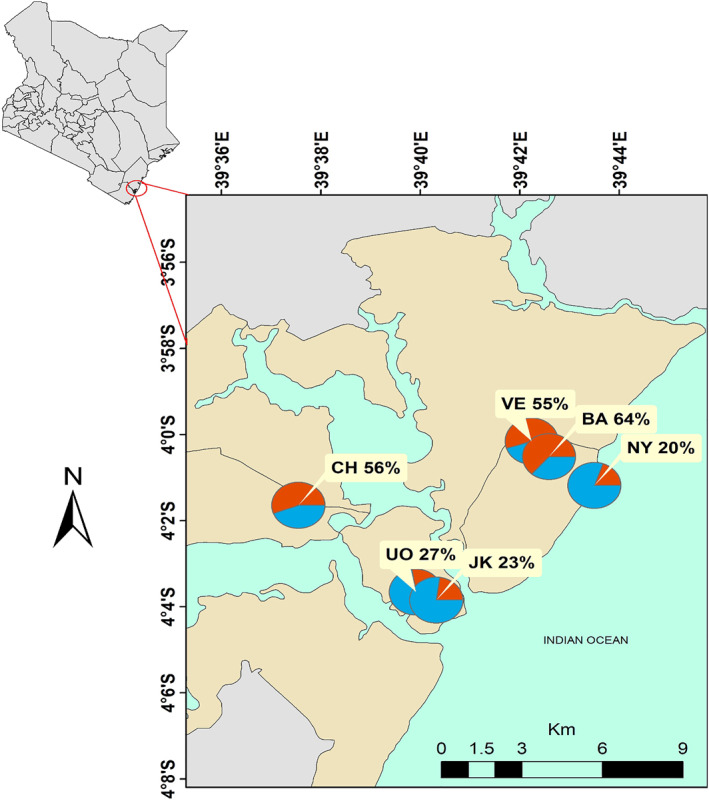
A map of Mombasa and the deployment sites of the Clarity Nodes and the tapered element oscillating microbalance. The pie charts show the percentage of days where the concentration of PM_2.5_ at each site exceeded (red) the WHO daily limit (15 μg m^−3^).

To gain a comprehensive understanding of air quality in Mombasa, this study focused on five distinct sampling locations in Changamwe, Vescon, Bamburi, the University of Nairobi (UoN), Jomo Kenyatta University of Agriculture and Technology (JKUAT) and Nyali (Figure 1). These locations (coordinates in Table 1) were strategically chosen to capture the diverse environmental conditions and potential sources of pollution within the city.

**Table 1:** Sensor Deployment Locations in Mombasa

Site	Site code	Latitude	Longitude	Description
Changamwe	CH	−4.027	39.626	Industrial near port
Vescon	VE	−4.003	39.704	Industrial site
Bamburi	BA	−4.009	39.710	Industrial and residential site
UON	UO	−4.061	39.665	Urban site
JKUAT	JK	−4.064	39.672	Ocean‐influenced
Nyali	NY	−4.020	39.725	Suburban residential area and ocean‐influenced

### 2.2 Clarity Node‐S

Clarity Node‐S (Clarity Movement Co., Berkeley, CA, USA) is a low‐cost multipollutant monitor that consists of a Plantower PMS6003, an electrochemical cell sensor (Alphasense), and a Bosche BME280 sensor for the simultaneous measurement of PM, NO_2_, temperature, and relative humidity (Nobell et al., 2023). The Plantower PMS6003 sensors are specifically designed for the measurement of PM and are equipped with two dual lasers that operate alternately, providing continuous cross‐verification to ensure sensor longevity (Nobell et al., 2023). When the sensor draws ambient air containing particles of different sizes into its measurement volume, a laser beam illuminates these particles. The resulting scattered light is then detected perpendicularly by a photodiode detector. Subsequently, the raw light signals undergo filtering and amplification through electronic filters and circuitry before being converted into mass concentrations. According to the manufacturer's data sheet, this particular sensor model has a measurement range spanning from 0.3 to 10 μm (Demanega et al., 2021; Kaur & Kelly, 2023), though laboratory studies have found that the Plantower PMS6003 and similar sensors have no ability to detect supermicron particles (Molina Rueda et al., 2023).

### 2.3 TEOM

The TEOM 1400a is a gravimetric PM monitor with the ability to make continuous mass measurements. It is one of the devices that has been designated as a Federal Equivalent Method by the United States Environmental Protection Agency. In principle, particle‐laden air streams are drawn through a filter medium weighed in near real‐time, usually every 2 s. The filter is placed on an elastic hollow glass‐like tube (the tapered element), free on one end but clamped on the other, and set in constant oscillation by an electronic feedback system. This motion has a light‐blocking effect on an LED‐phototransistor pair and can be used to detect the frequency of oscillation of the element. As more particles are deposited on the filter, this frequency decreases and the changes are converted into a mass measurement (Ardon‐Dryer et al., 2020; Kulkarni et al., 2011).

Changamwe, being an industrial area and home to the city's port activities, represents a hotspot for various industrial emissions. Vescon, situated in proximity to manufacturing and processing facilities, provides insights into the impact of industrial operations on air quality. Bamburi, with its mix of residential and industrial zones, serves as a representative sampling point for urban air quality. Nyali, a residential and tourist‐centric area with scenic beaches, contributes information on air quality in areas frequented by residents and visitors.

The UoN site serves as a reference point, providing data on air quality in an educational and research setting. It houses the reference monitor (TEOM) and one of the low‐cost sensors used in this study. The location at JKUAT has close proximity to the coastline and raises the possibility of sea spray contributing to local air quality dynamics. This is also true for Nyali found along the North coast of Mombasa. Each location offers a unique perspective on the challenges faced by Mombasa in maintaining air quality standards amid its economic and industrial activities.

### 2.4 Calibration Models

We collocated one Clarity Node‐S with a reference‐grade ThermoFisher TEOM 1400a installed at the UoN site from February to April 2023, spanning dry and wet months to fully account for seasonality. We compared the PM_2.5_ data from these devices using a univariate regression model similar to Badura et al., 2019, a multiple linear regression (MLR), a Gaussian Mixture Regression (GMR), and a random forest (RF) model similar to approaches followed by Malings et al. (2019) and McFarlane, Isevulambire, et al. (2021), McFarlane, Raheja, et al. (2021). These methods have been commonly used due to their ease of use (especially linear regression), their accuracy, and their frequency of use in the literature. Other correction models such as extreme gradient boosting, neural networks, or other machine learning approaches have been used as well (Giordano et al., 2021). The best performing correction model with respect to the *R* ^2^ and mean absolute error (MAE) values was retrospectively applied to a five‐sensor network in Mombasa that was operated between July 2021 and May 2022 to provide an overall survey of the air quality data in the city.

## 3 Results and Discussions

### 3.1 Correction of Low‐Cost Sensor Measurements

**Figure 2:**
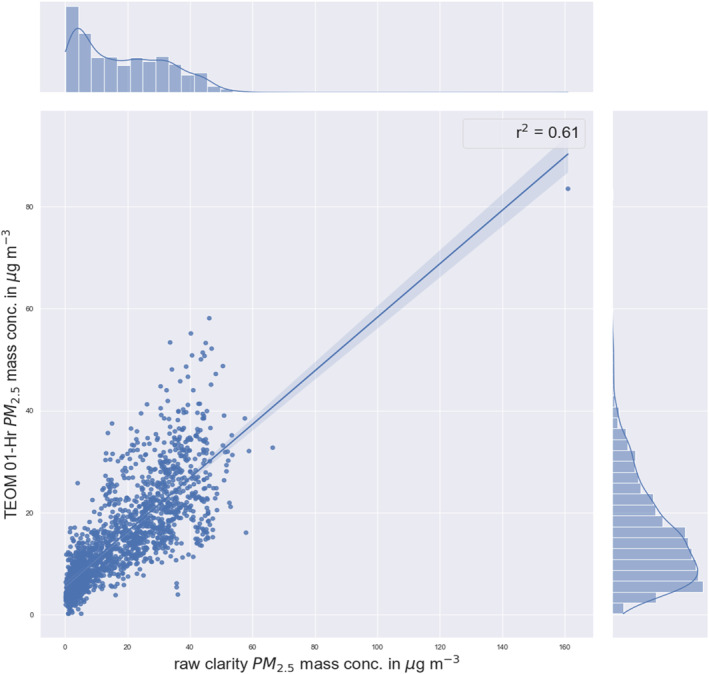
Performance evaluation and calibration of daily mean Clarity Node‐S against tapered element oscillating microbalance‐1400a PM_2.5_ data.

Figure 2 shows the daily averaged Clarity Node‐S PM_2.5_ data initial correlation with reference grade (TEOM) PM_2.5_ data with an *R* ^2^ value of 0.61 and initial mean absolute error (MAE = 7.03 μg m^−3^).

Including temperature and humidity data and modeling it using MLR, RF, and GMR models reduces the bias (Table 2). The MLR model had the best *R* ^2^ score of 0.61 and a reasonable MAE value of 4.28 μg m^−3^. Further statistical evaluation is shown in Table S2 in Supporting Information S1.

**Table 2:** The Statistical Performance Metrics of the Correction Models

Model	Statistical performance metrics	
	Coefficient of determination (*R* ^2^)	Mean absolute error (MAE) (μg m^−3^)
SLR	0.61	7.03
MLR	0.63	4.28
RF	0.60	4.40
GMR	0.44	3.93

**Figure 3:**
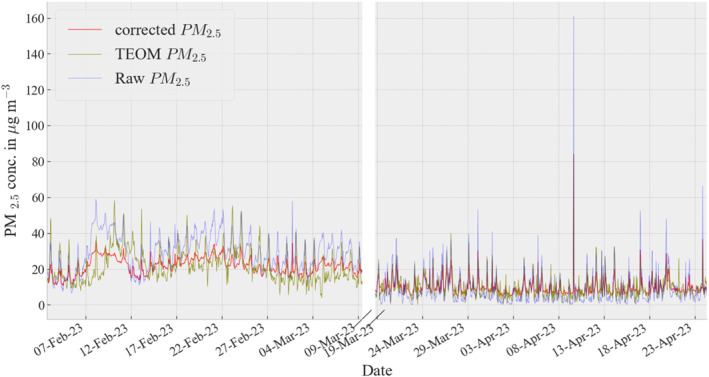
A time series plot displaying the corrected, Clarity Node‐S, and Tapered element oscillating microbalance‐1400a PM_2.5_ data.

Figure 3 shows the raw (purple), TEOM (olive), and corrected (red) hourly PM_2.5_ data collected at the UoN site from February to April 2023. On most days, the temporal trend was reproduced and the sensors responded well to sudden spikes of mass concentrations. However, the raw and reference data were within 10 μg m^−3^ in the month of March but within 20 μg m^−3^ in February. In addition, the daily averaged raw data of the Clarity Nodes in most cases overpredicted the concentrations compared to reference grade TEOM monitor during the co‐location period.

### 3.2 Daily PM_2.5_ Measurements

**Figure 4:**
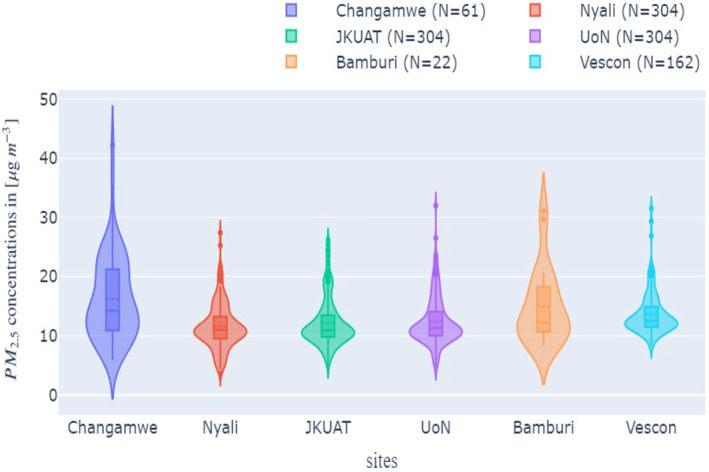
Violin plots of daily averaged corrected PM_2.5_ values for the entire data set at each location and six sites in Mombasa.

Figure 4 summarizes the daily means of corrected PM_2.5_ data from all six sites in a violin plot. Overall, the distributions are positively skewed mostly depicting a unimodal pattern and a majority of points between 10 and 20 μg m^−3^. Some sites like Changamwe and Vescon have long‐tail distributions compared to the rest, possibly alluding to heavy traffic or industrial activity experienced on some days. This is however not an exact intercomparison as different sites have different daily samples (indicated as N in the plots). According to the corrected plots, the highest daily PM_2.5_ values are observed in Changamwe (42 μg m^−3^) while the lowest daily concentrations are observed in Nyali (4 μg m^−3^). The average concentrations are also the highest and lowest at these sites with Changamwe recording daily average of 16 μg m^−3^ respectively while Nyali has average of 11 μg m^−3^ respectively. Only the daily average of Changamwe exceeded the WHO daily PM_2.5_ limit of 15 μg m^−3^ though there were days when this limit was exceeded in the other sites.

### 3.3 PM_2.5_ Time Series Plot at Each Site

**Figure 5:**
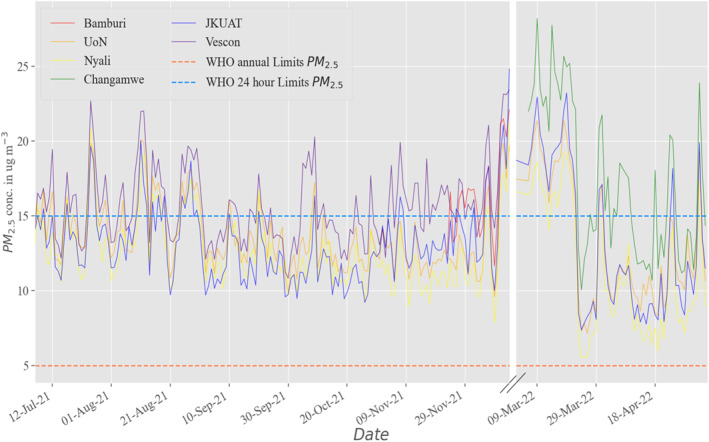
Timeseries plots of the daily PM_2.5_ concentrations in six sites in Mombasa from July 2021 to May 2022.

Figure 5 shows the temporal variations of corrected daily PM_2.5_ concentrations from the six sites in Mombasa. Overall, the concentrations at each site exceeds the WHO annual guidelines of 5.0 μg m^−3^ in all days and exceeded the daily limit of 15.0 μg m^−3^ on only some days, ranging from 20% to 64% of days depending on the location (see pie charts in Figure 1).

Seasonal variations in PM_2.5_ concentrations are evident with the highest monthly averages observed during the dry months (December to February) when the wet deposition is greatly reduced. This was followed by the cold months (July and August) where elevated PM_2.5_ averages are also consistent with the lack of precipitation during this time period. By comparison, the lowest averages were in April and between October and November which correspond to the wet months where washout effect of the rain and wet deposition reduce the PM_2.5_ levels.

### 3.4 Temporal Patterns in PM_2.5_ Concentrations

**Figure 6:**
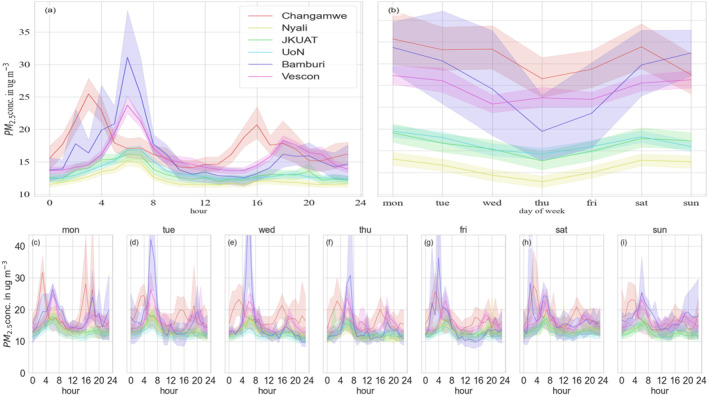
Hourly average PM_2.5_ concentrations of six sites in Mombasa organized into hour‐of‐day and day‐of‐week temporal trends. Shading represents the range.

The diurnal cycles, weekly, and daily variations of PM_2.5_ in the six sites in Mombasa are presented in Figure 6. The highest PM_2.5_ concentrations are most likely to appear on during weekends in a weekly cycle, and most unlikely to appear on Thursdays. The large increases in tourist activity and consequently motor vehicles in the weekends are likely to be a reason leading to elevated PM_2.5_ levels.

For five of the sites the diurnal cycles of PM_2.5_ (top‐left panel) displayed a bimodal pattern with early morning peaks between (6:00 a.m. and 8:00 a.m.) and afternoon peaks between (5.00 p.m. and 9:00 p.m.). This was consistent with the increased anthropogenic activity caused by commuter travel habits during rush hour times and also by the changing mixing height. This is with exception to Changamwe whose morning and evening peaks came in much earlier than the other sites, most likely because of the activities at the port. During the rest of the day, traffic activities reduce and there is more mixing of pollutants hence a decrease in PM_2.5_ concentrations.

One caveat of our study includes the retrospectively applied correction factor based on a single node air sensor and reference monitor co‐location for only a few months. While best practice suggests a more robust co‐location, similar approaches have been successfully applied in previous studies, especially in data sparse areas such as the African continent (McFarlane, Isevulambire, et al., 2021; Raheja et al., 2022). However, due to the timing of the colocation, we cover both a dry month (February) and wet months (March and April), thereby accounting for the predominant seasonality in the region. Additionally, concentrations during the colocation period months and the deployment months are similar in magnitude, suggesting that the co‐location period was, to first order, an appropriate proxy for the deployment period. Finally, the co‐location period had similar environmental conditions as the deployment period, as demonstrated in Table S1 in Supporting Information S1.

## 4 Conclusion and Recommendations

In conclusion, this study addresses the significant challenge of limited surface measurements of fine PM_2.5_ in Sub‐Saharan Africa, particularly in Mombasa, Kenya. The evaluation of Clarity Node‐S PM sensors against a TEOM revealed moderate correlation and a MAE of approximately 7.03 μg m^−3^ in raw, manufacturer‐reported data. Through the application of calibration models, including MLR, GMR, and RF, the MAE was reduced to 4.28, 3.93, and 4.40 μg m^−3^, respectively, with MLR achieving the highest *R* ^2^ value of 0.63.

Applying the correction factor to a five‐sensor network in Mombasa provided valuable insights into the air quality, revealing average daily PM_2.5_ concentrations ranging from 12 to 18 μg m^−3^. Some sites, such as Changamwe, Vescon, and Bamburi, exceeded WHO daily PM_2.5_ guidelines more than 50% of the time. Higher averages were observed during dry and cold seasons and during early morning and evening hours.

The study highlights the potential of low‐cost sensor systems in regions with limited monitoring infrastructure, emphasizing their role in providing reliable air quality data where reference monitors are scarce. The findings contribute to the nascent field of air quality research in Mombasa, offering valuable information for future interventions and policies aimed at mitigating the health risks associated with air pollution. Though additional investigation is needed with larger networks, our first results suggest that PM_2.5_ concentrations are moderately lower than other major African cities (e.g., Nairobi) (Pope et al., 2018). This could be attributed to many factors, likely including the close proximity to clean oceanic air masses owing to Mombasa's coastal location. The temporal and spatial variations in PM_2.5_ concentrations underscore the need for continuous monitoring and targeted interventions to address air quality challenges in LMICs like Kenya. Future research should explore other areas within the city or other air pollutants not yet explored. Satellite data can also be used to map out potential hotspots followed by dedicated studies looking at the sources of pollution in the city.

## Conflict of Interest

The authors declare no conflicts of interest relevant to this study.

## Supporting information

Supporting Information S1

## Data Availability

All data and scripts used in this project are available on the Zenodo repository, which follows FAIR data guidelines (Westervelt, 2024).

## References

[gh2574-bib-0001] Alfano, B. , Barretta, L. , Del Giudice, A. , De Vito, S. , Di Francia, G. , Esposito, E. , et al. (2020). A review of low‐cost particulate matter sensors from the developers’ perspectives. Sensors, 20(23), 6819. 10.3390/s20236819 33260320 PMC7730878

[gh2574-bib-0002] Amegah, A. K. (2018). Proliferation of low‐cost sensors. What prospects for air pollution epidemiologic research in Sub‐Saharan Africa? Environmental Pollution, 241, 1132–1137. 10.1016/j.envpol.2018.06.044 30029322

[gh2574-bib-0003] Ardon‐Dryer, K. , Dryer, Y. , Williams, J. N. , & Moghimi, N. (2020). Measurements of PM_2.5_ with PurpleAir under atmospheric conditions. Atmospheric Measurement Techniques, 13(10), 5441–5458. 10.5194/amt-13-5441-2020

[gh2574-bib-0004] Badura, M. , Batog, P. , Drzeniecka‐Osiadacz, A. , & Modzel, P. (2019). Regression methods in the calibration of low‐cost sensors for ambient particulate matter measurements. SN Applied Sciences, 1(6), 622. 10.1007/s42452-019-0630-1

[gh2574-bib-0005] Demanega, I. , Mujan, I. , Singer, B. C. , Anđelković, A. S. , Babich, F. , & Licina, D. (2021). Performance assessment of low‐cost environmental monitors and single sensors under variable indoor air quality and thermal conditions. Building and Environment, 187, 107415. 10.1016/j.buildenv.2020.107415

[gh2574-bib-0006] Dharaiya, V. R. , Malyan, V. , Kumar, V. , Sahu, M. , Venkatraman, C. , Biswas, P. , et al. (2023). Evaluating the performance of low‐cost PM sensors over multiple COALESCE network sites. Aerosol and Air Quality Research, 23(5), 220390. 10.4209/aaqr.220390

[gh2574-bib-0007] Feenstra, B. , Papapostolou, V. , Hasheminassab, S. , Zhang, H. , Boghossian, B. D. , Cocker, D. , & Polidori, A. (2019). Performance evaluation of twelve low‐cost PM2.5 sensors at an ambient air monitoring site. Atmospheric Environment, 216, 116946. 10.1016/j.atmosenv.2019.116946

[gh2574-bib-0008] Ghamari, M. , Soltanpur, C. , Rangel, P. , Groves, W. A. , & Kecojevic, V. (2022). Laboratory and field evaluation of three low‐cost particulate matter sensors. IET Wireless Sensor Systems, 12(1), 21–32. 10.1049/wss2.12034

[gh2574-bib-0009] Ghosh, R. , Causey, K. , Burkart, K. , Wozniak, S. , Cohen, A. , & Brauer, M. (2021). Ambient and household PM2.5 pollution and adverse perinatal outcomes: A meta‐regression and analysis of attributable global burden for 204 countries and territories. PLoS Medicine, 18(9), e1003718. 10.1371/journal.pmed.1003718 34582444 PMC8478226

[gh2574-bib-0010] Giordano, M. R. , Malings, C. , Pandis, S. N. , Presto, A. A. , McNeill, V. F. , Westervelt, D. M. , et al. (2021). From low‐cost sensors to high‐quality data: A summary of challenges and best practices for effectively calibrating low‐cost particulate matter mass sensors. Journal of Aerosol Science, 158, 105833. 10.1016/J.JAEROSCI.2021.105833

[gh2574-bib-0011] Hagan, D. H. , & Kroll, J. H. (2020). Assessing the accuracy of low‐cost optical particle sensors using a physics‐based approach. Atmospheric Measurement Techniques, 13(11), 6343–6355. 10.5194/amt-13-6343-2020 33777248 PMC7995643

[gh2574-bib-0012] Hoffmann, B. , Boogaard, H. , de Nazelle, A. , Andersen, Z. J. , Abramson, M. , Brauer, M. , et al. (2021). WHO air quality guidelines 2021—Aiming for healthier air for all: A joint statement by Medical, Public health, scientific societies and patient representative organisations. International Journal of Public Health, 66, 1604465. 10.3389/ijph.2021.1604465 34630006 PMC8494774

[gh2574-bib-0013] Jayaratne, R. , Liu, X. , Thai, P. , Dunbabin, M. , & Morawska, L. (2018). The influence of humidity on the performance of a low‐cost air particle mass sensor and the effect of atmospheric fog. Atmospheric Measurement Techniques, 11(8), 4883–4890. 10.5194/amt-11-4883-2018

[gh2574-bib-0014] Kaur, K. , & Kelly, K. E. (2023). Laboratory evaluation of the Alphasense OPC‐N3, and the Plantower PMS5003 and PMS6003 sensors. Journal of Aerosol Science, 171, 106181. 10.1016/j.jaerosci.2023.106181

[gh2574-bib-0015] Kenyan National Bureau of Statistics . (2019). 2019 Kenya population and housing census: Volume II: Distribution of population by administrative units. Republic of Kenya.

[gh2574-bib-0016] KPA . (2017). Environmental and social impact assessment study report for Rehabilitation of Berths 1‐14.

[gh2574-bib-0017] Kulkarni, P. , Baron, P. A. , & Willeke, K. (2011). Aerosol measurement: Principles, techniques, and applications (3rd ed.). Wiley. Retrieved from http://gen.lib.rus.ec/book/index.php?md5=051eda4f5298dbb0b039d8426e664ed2

[gh2574-bib-0018] Levy Zamora, M. , Xiong, F. , Gentner, D. , Kerkez, B. , Kohrman‐Glaser, J. , & Koehler, K. (2019). Field and laboratory evaluations of the low‐cost plantower particulate matter sensor. Environmental Science & Technology, 53(2), 838–849. 10.1021/acs.est.8b05174 30563344

[gh2574-bib-0019] Malings, C. , Tanzer, R. , Hauryliuk, A. , Kumar, S. P. N. , Zimmerman, N. , Kara, L. B. , et al. (2019). Development of a general calibration model and long‐term performance evaluation of low‐cost sensors for air pollutant gas monitoring. Atmospheric Measurement Techniques, 12(2), 903–920. 10.5194/amt-12-903-2019

[gh2574-bib-0020] McDuffie, E. , Martin, R. , Yin, H. , & Brauer, M. (2021). Global burden of disease from major air pollution sources (GBD MAPS): A global approach. Research Report (Health Effects Institute), 2021(210), 1–45. PMC9501767 36148817

[gh2574-bib-0021] McFarlane, C. , Isevulambire, P. K. , Lumbuenamo, R. S. , Ndinga, A. M. E. , Dhammapala, R. , Jin, X. , et al. (2021). First measurements of ambient PM2.5 in Kinshasa, democratic Republic of Congo and Brazzaville, Republic of Congo using field‐calibrated low‐cost sensors. Aerosol and Air Quality Research, 21(7), 200619. 10.4209/AAQR.200619

[gh2574-bib-0022] McFarlane, C. , Raheja, G. , Malings, C. , Appoh, E. K. E. , Hughes, A. F. , & Westervelt, D. M. (2021). Application of Gaussian mixture regression for the correction of low cost PM2.5 monitoring data in Accra, Ghana. ACS Earth and Space Chemistry, 5(9), 2268–2279. 10.1021/acsearthspacechem.1c00217

[gh2574-bib-0023] Molina Rueda, E. , Carter, E. , L’Orange, C. , Quinn, C. , & Volckens, J. (2023). Size‐resolved field performance of low‐cost sensors for particulate matter air pollution. Environmental Science and Technology Letters, 10(3), 247–253. 10.1021/acs.estlett.3c00030 36938150 PMC10018765

[gh2574-bib-0024] Nobell, S. , Majumdar, A. , Mukherjee, S. , Chakraborty, S. , Chatterjee, S. , Bose, S. , et al. (2023). Validation of in‐field calibration for low‐cost sensors measuring ambient particulate matter in Kolkata, India. Aerosol and Air Quality Research, 23(11), 230010. 10.4209/aaqr.230010

[gh2574-bib-0025] Okure, D. , Ssematimba, J. , Sserunjogi, R. , Gracia, N. L. , Soppelsa, M. E. , & Bainomugisha, E. (2022). Characterization of ambient air quality in selected urban areas in Uganda using low‐cost sensing and measurement technologies. Environmental Science & Technology, 56(6), 3324–3339. 10.1021/ACS.EST.1C01443/ASSET/IMAGES/LARGE/ES1C01443_0015.JPEG 35147038

[gh2574-bib-0026] Ouimette, J. R. , Malm, W. C. , Schichtel, B. A. , Sheridan, P. J. , Andrews, E. , Ogren, J. A. , & Arnott, W. P. (2022). Evaluating the PurpleAir monitor as an aerosol light scattering instrument. Atmospheric Measurement Techniques, 15(3), 655–676. 10.5194/AMT-15-655-2022

[gh2574-bib-0027] Pope, F. D. , Gatari, M. , Ng'ang'a, D. , Poynter, A. , & Blake, R. (2018). Airborne particulate matter monitoring in Kenya using calibrated low‐cost sensors. Atmospheric Chemistry and Physics, 18(20), 15403–15418. 10.5194/acp-18-15403-2018

[gh2574-bib-0028] Raheja, G. , Nimo, J. , Appoh, E. K.‐E. , Essien, B. , Sunu, M. , Nyante, J. , et al. (2023). Low‐cost sensor performance intercomparison, correction factor development, and 2+ years of ambient PM2.5 monitoring in Accra, Ghana. Environmental Science and Technology, 75(29), 10708–10720. 10.1021/acs.est.2c09264 PMC10373484 37437161

[gh2574-bib-0029] Raheja, G. , Sabi, K. , Sonla, H. , Gbedjangni, E. K. , McFarlane, C. M. , Hodoli, C. G. , & Westervelt, D. M. (2022). A network of field‐calibrated low‐cost sensor measurements of PM2.5 in Lomé, Togo, over one to two years. ACS Earth and Space Chemistry, 6(4), 1011–1021. 10.1021/acsearthspacechem.1c00391 35495364 PMC9036579

[gh2574-bib-0030] Simiyu, A. H. , Muthama, J. , Ngaina, J. , & Onwonga, R. (2018). Anthropogenic contribution to air pollution with background emissions; case of Nairobi, Mombasa and Kisumu. International Journal of Scientific and Research Publications (IJSRP), 8(8), 8047. 10.29322/IJSRP.8.8.2018

[gh2574-bib-0031] Subramanian, R. , & Garland, R. M. (2021). The powerful potential of low‐cost sensors for air quality research in Africa. Clean Air Journal, 31(1), 1‐1. 10.17159/CAJ/2021/31/1.11274

[gh2574-bib-0032] Tryner, J. , L’Orange, C. , Mehaffy, J. , Miller‐Lionberg, D. , Hofstetter, J. C. , Wilson, A. , & Volckens, J. (2020). Laboratory evaluation of low‐cost PurpleAir PM monitors and in‐field correction using co‐located portable filter samplers. Atmospheric Environment, 220, 117067. 10.1016/j.atmosenv.2019.117067

[gh2574-bib-0033] Westervelt, D. M. (2024). Mombasa TEOM and Clarity network data [Dataset]. Zenodo. 10.5281/zenodo.10836211

[gh2574-bib-0034] Westervelt, D. M. , Isevulambire, P. K. , Yombo Phaka, R. , Yang, L. H. , Raheja, G. , Milly, G. , et al. (2024). A Low cost investigation into sources of PM2.5 in Kinshasa, DRC. ACS EST Air, 1(1), 43–51. 10.1021/acsestair.3c00024

[gh2574-bib-0035] Yussuf, E. , Muthama, J. N. , Mutai, B. , & Marangu, D. M. (2023). Impacts of air pollution on pediatric respiratory infections under a changing climate in Kenyan urban cities. East African Journal of Science, Technology and Innovation, 4, 2. 10.37425/eajsti.v4i2.579

[gh2574-bib-0036] Zimmerman, N. , Presto, A. A. , Kumar, S. P. N. , Gu, J. , Hauryliuk, A. , Robinson, E. S. , et al. (2018). A machine learning calibration model using random forests to improve sensor performance for lower‐cost air quality monitoring. Atmospheric Measurement Techniques, 11(1), 291–313. 10.5194/amt-11-291-2018

