# Supplementary material for: First Results From a Calibrated Network of Low‐Cost PM2.5 Monitors in Mombasa, Kenya Show Exceedance of Healthy Guidelines
Source: Geohealth. 2024 Sep 21;8(9):e2024GH001049. doi: 10.1029/2024GH001049 (PMC11415614; doi:10.1029/2024GH001049)
Supplement: Supplementary file 1 — Supporting Information S1 [file GH2-8-e2024GH001049-s001.docx]

Supporting information for…

First Results from a Calibrated Network Low-Cost PM_2.5_ Monitors in Mombasa, Kenya Show Exceedance of Healthy Guidelines

M. N. Njeru^1^, E. Mwangi^1^, M. J. Gatari^1^, M. I. Kaniu^2^, J. Kanyeria^3^, G. Raheja^4,5^, D. M. Westervelt^5^

^1^Institute of Nuclear Science and Technology, University of Nairobi, Kenya.

^2^Department of Physics, University of Nairobi, Kenya.

^3^Institute of Energy and Environmental Technology, Jomo Kenyatta University of Agriculture and Technology, Kenya.

^4^Department of Earth and Environmental Sciences, Columbia University, New York, NY, USA

^5^Lamont-Doherty Earth Observatory of Columbia University, New York, NY, USA

Table S1. Meteorological data comparison between the sampling period and co-location period using Modern-Era Restrospective analysis for Research and Applications, Version 2 (MERRA-2)

| period | Temperature_2m (^0^C) | Relative Humidity (%) | WD_2M (^o^) | WS_2M (m s^-1^) | cardinal_WD |
| --- | --- | --- | --- | --- | --- |
| Collocation Period | 27.67 | 78.38 | 40.5 | 2.26 | NE |
| Sampling Period | 26.76 | 77.12 | 13.99 | 3.02 | NNE |

Table S2. Statistical performance metrics of each of the correction models. See Giordano et al. (2021) for equations for statistical parameters.

| Model | Statistical Performance Metrics | | | |
| --- | --- | --- | --- | --- |
|  | Coefficient of Determination (R^2^) | Cv  MAE  (μg m^–3^) | MAE  (μg m^–3^) | nRMSE |
| As-reported | 0.61 | 0.46 | 7.03 | 5.34 |
| MLR | 0.63 | 0.27 | 4.28 | 2.5 |
| RF | 0.60 | 0.28 | 4.40 | 2.4 |
| GMR | 0.44 | 0.26 | 3.93 | 2.0 |
